# RNA Biology Provides New Therapeutic Targets for Human Disease

**DOI:** 10.3389/fgene.2019.00205

**Published:** 2019-03-08

**Authors:** Lorna W. Harries

**Affiliations:** RNA-Mediated Mechanisms of Disease, College of Medicine and Health, The Institute of Biomedical and Clinical Science, Medical School, University of Exeter, Exeter, United Kingdom

**Keywords:** mRNA processing, RNA editing, RNA export, RNA therapeutics, ncRNA, splicing, RNA epitranscriptomics, therapeutics

## Abstract

RNA is the messenger molecule that conveys information from the genome and allows the production of biomolecules required for life in a responsive and regulated way. Most genes are able to produce multiple mRNA products in response to different internal or external environmental signals, in different tissues and organs, and at specific times in development or later life. This fine tuning of gene expression is dependent on the coordinated effects of a large and intricate set of regulatory machinery, which together orchestrate the genomic output at each locus and ensure that each gene is expressed at the right amount, at the right time and in the correct location. This complexity of control, and the requirement for both sequence elements and the entities that bind them, results in multiple points at which errors may occur. Errors of RNA biology are common and found in association with both rare, single gene disorders, but also more common, chronic diseases. Fortunately, complexity also brings opportunity. The existence of many regulatory steps also offers multiple levels of potential therapeutic intervention which can be exploited. In this review, I will outline the specific points at which coding RNAs may be regulated, indicate potential means of intervention at each stage, and outline with examples some of the progress that has been made in this area. Finally, I will outline some of the remaining challenges with the delivery of RNA-based therapeutics but indicate why there are reasons for optimism.

## Introduction

The fundamental importance of RNA not only as a messenger molecule, but as a regulator of genes in its own right is increasingly being recognized. The production of mature messenger RNA (mRNA) is dependent on a plethora of processing and regulatory steps involving a complicated repertoire of sequence elements, RNA binding proteins and other regulatory RNA species. Given the complexity of the regulatory machinery, defects in non-coding regions of genes and regulatory genomic regions are common in genetic disease, being present in up to 50% of cases (Yang et al., 2013; Beaulieu et al., 2014) and are also the most common site of genetic variation conferring susceptibility to common, complex disease (Manolio et al., 2008). There is, however, a silver lining. The complexity that causes errors in gene expression or mRNA processing to be such a common occurrence, also provides multiple and differential points of potential therapeutic intervention. Over the past decade, there have been a number of examples, where the specifics of RNA regulatory machinery have been harnessed to produce novel therapeutics that are now in phase III clinical trials [e.g., Patisiran for Familial amyloid polyneuropathy (Rizk and Tuzmen, 2017), Custirsen for prostate cancer (Edwards et al., 2017) and AGS-003 for renal cell carcinoma (Figlin, 2015)]. This review aims to explore the potential for intervention in mRNA processing or post-transcriptional regulation with selected examples for future therapeutic benefit.

## The Lifecycle of a Coding RNA

**FIGURE 1:**
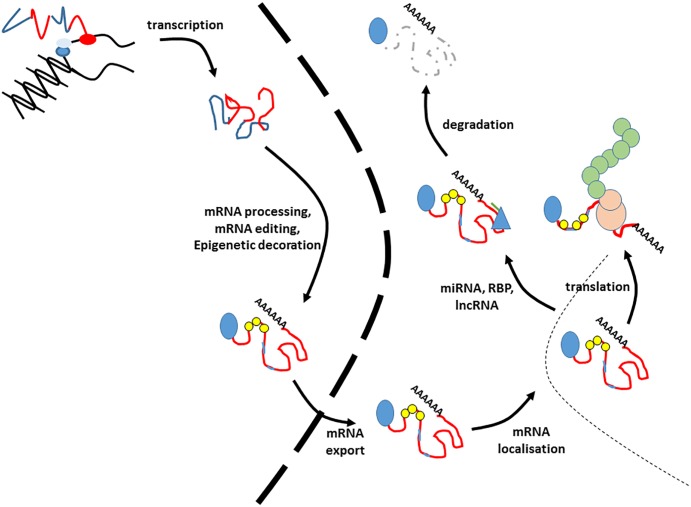
The lifecycle of an RNA. This figure illustrates the lifecycle of an mRNA. These processes are co-transcriptional, so the precise order of events is illustrative. Blue lines in the transcript refer to introns and untranslated regions, whilst exons are indicted by red lines. The 5′ cap is indicated by a blue circle. Small yellow circles indicate epitranscriptomic decoration, whilst pale blue lines within the exons refer to RNA editing events. The nuclear envelope is indicated by a large dashed line. RNA binding proteins modifying stability are given by blue triangles, and miRNAs by green lines. The translating ribosome is indicated by beige circles. Nascent polypeptide is given by green interlocked circles. Degraded RNA is indicated by a gray dashed line.

The processes involved in the production of a mature mRNA, and its subsequent fate are multifaceted and complicated (Figure 1). The life of an RNA molecule starts upon transcription, which is controlled by tissue specific promoters and enhancers. The immature primary RNA transcript (heterogeneous nuclear RNA (hnRNA) or pre-mRNA) then undergoes a series of modifications that involve the addition of the 5′ cap structure, removal of the intronic sequences by constitutive or alternative splicing and 3′ end processing events that include the addition of the poly-A tail (Chen et al., 2017; Sperling, 2017; Zhang and Tjian, 2018). These processes are not a linear pipeline and occur co-transcriptionally (Beyer and Osheim, 1988; Bentley, 2002). Newly processed RNA may also undergo RNA editing, which is mostly A to G or A to I substitution in humans (Chen, 2013). RNAs may also undergo epitranscriptomic decoration, whereby different RNA modifications such as methylation of adenosine residues (m^6^A) may be added. Such modifications are added by a series of RNA readers, writers and erasers (Helm and Motorin, 2017). Mature mRNAs are then exported from the nucleus to the cytoplasm. This is an active and regulated process, and one of the primary safeguards against the translation of aberrant mRNAs (Williams et al., 2018). The spatial and temporal expression of newly exported RNAs can also controlled at the level of specific localization within the cell. This can be passive, or an active process involving transport on cytoskeletal tracks (Suter, 2018). Gene expression can also be controlled at the level of translation. This can occur by virtue of selective degradation of specific RNAs by mRNA surveillance pathways such as nonsense-mediated decay, no-go decay and non-stop decay (Harigaya and Parker, 2010; Klauer and van Hoof, 2012; Lejeune, 2017), or it can be by regulation of the rate of translation itself (Gorgoni et al., 2014). The half-life of any given mRNA is then determined by a number of RNA decay pathways, most of which involve successive decapping and deadenylation of RNA molecules, which then renders them susceptible to exonucleases (Wahle and Winkler, 2013; Borbolis and Syntichaki, 2015). Finally, the fate of the RNA may also be influenced by the action of both short and long non-coding RNAs and RNA binding proteins which can result in degradation or translational blocking (Fukao et al., 2015; Iadevaia and Gerber, 2015; Fukao and Fujiwara, 2017).

## Potential Points of Therapeutic Intervention

Knowledge of the processes by which mature mRNAs are expressed, processed and regulated opens up the possibility of targeting the molecule with specific interventions for future therapeutic benefit.

### Therapeutic Modulation of Transcription

Therapeutic modulation of gene activity can be achieved through several mechanisms which include triplex-forming oligonucleotides (TPOs) synthetic polyamides (SPs) and artificial transcription factors (ATFs) (Uil et al., 2003). These approaches work by altering the expression level of a gene, rather than restoring its sequence *per se*. TPOs and SPs work by binding the major and minor groove, respectively, of the genomic DNA in specific regions of the gene, with the consequence of modulating gene activity at the level of transcription. This can be achieved by using steric hindrance to block transcription elongation for down-regulation of gene activity or conversely, blocking access to naturally occurring repressor molecules to bring about gene activation. ATFs are custom molecules designed with DNA binding domains specific to the gene in question, coupled to a *tran*s-regulatory domain to produce the desired activity. Although there have been some promising *in vitro* studies, such as reactivation of the *EPB41L3* gene, usually silenced by methylation, to promote tumor suppression in breast, ovarian, and cervical cell lines (Huisman et al., 2015), they have not yet reached prominence in the clinic.

### Therapeutic Modification of Splicing

RNA splicing is controlled by a complex interplay between ribonucleoprotein complexes and sequence elements in the pre-mRNA. The splicing process consists of two phosphodiester transfer reactions; the first being an interaction between the 5′ splice site and the branch site, and the second comprising cleavage at the 3′ splice site, and joining of the released exons. This occurs due to the action of a family of small nuclear ribonucleoproteins (snRNPs) named U1, U2, U4, U5, and U6, which together with a battery of approximately 80 other ancillary proteins form the core spliceosome and orchestrate the splicing process (Will and Luhrmann, 2011). The spliceosome is a dynamic machine that undergoes structural remodeling and conformational change to bring about the excision of introns and the joining of introns (Makarov et al., 2002). This machinery is necessary but sometimes not sufficient for splice site usage to occur; 98% of the genome produces multiple RNA transcripts in a process termed alternative splicing (Pan et al., 2008). The precise nature of transcripts produced under different circumstances is under tight spatial and temporal regulation. This is facilitated by the combinatorial control of a series of splice site activators and inhibitor proteins that together determine whether or not a given splicing event occurs in a given circumstance. Serine Arginine rich proteins (SRSF) splicing factors usually (but not exclusively) promote splice site usage, whereas heterogeneous nuclear ribonucleoproteins (hnRNPs) usually (but not exclusively) promote splice site silencing, as well as having roles in nuclear export and other aspects of RNA metabolism (Smith and Valcarcel, 2000; Cartegni et al., 2002). Splicing defects can arise from single base pair changes to the core and regulatory sequence elements, but can also arise from insertion or deletion events and frameshifts, or from activation of cryptic splice sites by other sequence changes. Similarly, changes occurring in exon and intron splicing enhancer and silencer elements can elicit dysregulation of splicing patterns of specific genes (Blencowe, 2000). Dysregulation of the splicing regulatory machinery by cellular stress has been reported in more complex phenotypes such as cellular senescence (Holly et al., 2013; Latorre et al., 2017) and altered global alternative splicing profiles are a key characteristics of many complex diseases such as dementia, cancer and type 2 diabetes (Tollervey et al., 2011; Berson et al., 2012; Cnop et al., 2014; Love et al., 2015; Lu et al., 2015). The complexity of splicing regulation offers several points of potential intervention.

#### Moderation of the Core Spliceosome

The global dysregulation of splicing patterns that occur in complex disease may be addressed by targeting the core spliceosome. There are several compounds of bacterial origin that affect the function of the SF3B component of the U2 snRNP, which are showing promise as anti-cancer agents by causing stalling of the cell cycle at the G1/S or G2/M checkpoints (Nakajima et al., 1996). Although these approaches show promise, to date most remain some distance from the clinic.

#### Moderation of Splicing Regulation

It may be possible to globally restore splicing patterns by targeting the splicing regulatory proteins themselves. This could be done at the level of mRNA expression, or at the level of activation or cellular localization. Splicing factor expression has recently been described to be negatively regulated at the mRNA level in senescent primary human dermal fibroblasts by the constitutive activation of the ERK and AKT pathways. Targeted inhibition of either ERK or AKT, as well as gene knock down of their effector genes *FOXO1* and *ETV6* was associated with restoration of splicing factor expression and rescue from cellular senescence (Latorre et al., 2018). Similarly, splicing factor activity and localization is controlled at the protein level by the action of a series of kinases and phosphatases including SRPK1, SRPK2, CLK1 - CLK4, DYRK1-2, PIM1-2, and PRP4. The action of these regulators ensures the correct localization of splicing factors for action at the correct time and in the correct place. Several small molecule inhibitors of SRPK1 or SRPK2 are in development currently and show promise as anti-cancer agents for prostate malignancy in humans (Mavrou et al., 2015; Bates et al., 2017). Similarly, CLK protein kinase inhibitors have been demonstrated to suppress cell growth in human mammary tumor cell lines (Araki et al., 2015).

#### Moderation of Splice Site Choice

If monogenic disease is due to dysregulated splicing, in some cases it may be possible to correct or reverse the defect by restoration of correct splicing patterns. There are several means of accomplishing this, including antisense oligonucleotides (AONs), or steric hindrance agents such as morpholino oligonucleotides or similar to occlude specific splicing regulatory sequences. This potential of this approach is best exemplified by novel treatments for spinal muscular atrophy (SMA) and Duchenne Muscular dystrophy (DMD) for which therapies for manipulation of splicing have been developed and are now licensed for clinical use. SMA is characterized by progressive neuromuscular disorder caused by mutations in the Survival Motor Neuron (*SMN1*) gene (Lefebvre et al., 1995; Lorson et al., 1999). These are often deletion events. The human genome contains a second SMN gene, *SMN2*, which due to the presence of a single C-to-T transition at codon 280 which disrupts a splicing enhancer site produces an unstable *SMN* transcript lacking exon 7 (SMNΔ7). This transcript is present at only 10% of *SMN1* levels (Lorson et al., 1999) but has potential to compensate for mutation-related reduced activity of *SMN1*. This has formed the basis for a novel therapeutic strategy whereby an AON (Nusinersen) has been designed to influence splicing patterns of *SMN2*. Nusinersen targets the N1 (ISS-N1) motif in *SMN2*, and promotes the inclusion of exon 7 and increases levels of compensatory *SMN2*. Several clinical trials have now been undertaken (Parente and Corti, 2018) and Nusinersen, also known as Spinraza, has now been approved by both US and EU regulatory authorities for clinical use.

Similar strategies have also been employed for Duchenne Muscular dystrophy, an X-linked neuromuscular disorder that affects 1:5000 newborn boys (Mendell et al., 2012), and is primarily caused by deletions, frameshift or nonsense mutations in the dystrophin (*DMD*) gene (Monaco et al., 1988). The majority of these mutations yield mRNAs containing premature termination codons, which trigger nonsense-mediated decay and degradation of affected *DMD* transcripts. Several strategies involving AONs targeted to specific splice sites have now been employed to bring about exon skipping to remove the offending exon(s) and lead to the production of a truncated, but still partially functional DMD protein (Aartsma-Rus, 2010; Niks and Aartsma-Rus, 2017). Similar approaches have been employed to modify the effects of duplication mutations in cell lines (Wein et al., 2017). Most AONs under assessment as DMD therapeutics are chemically modified 2′-*O*-methyl-phosphorothioate oligonucleotides (2OMePS) or phosphorodiamidate morpholino oligomers (PMOs) which can be administered systemically (Goemans et al., 2011). One of these, eteplirsen, a PMO which brings about skipping of exon 51, a hotspot for DMD mutations, has demonstrated promising results in a number of clinical trials and been designated ‘reasonably likely to predict a clinical benefit’ by the FDA (Goemans et al., 2011). Other approaches have employed ‘readthrough’ agents such as ataluren that allow bypass of the premature termination codon and are now in Phase III clinical trials (Namgoong and Bertoni, 2016).

### Therapeutic Moderation of Polyadenylation

Polyadenylation is an essential step in mRNA processing, with a pivotal role in maintenance of RNA stability and management of RNA turnover. Many genes contain more than one polyadenylation site and display alternative polyadenylation, producing mRNA transcripts with novel 3′ untranslated regions. These may be differentially targeted by non-coding RNAs such as miRNAs or RNA binding proteins, or have differential translation efficiency (Elkon et al., 2013). Control of polyadenylation is mediated by a number of sequence elements such as the polyadenylation site itself, but also a series of upstream (U and UGUA rich) and downstream (U and GU rich) elements (Tian and Graber, 2012) that bind the protein complexes that orchestrate the process. These sequence elements bind the polyadenylation machinery that include the cleavage and polyadenylation specificity factors, the cleavage stimulation factors and the polyadenylate polymerase itself (Shi et al., 2009). Differential choice of polyadenylation site is linked to the proliferation and differentiation capacity of the cells; transcripts in highly proliferative cells tend to have shorter 3′UTRs (Sandberg et al., 2008). Differential use of polyadenylation sites may also have impacts on mRNA stability, mRNA export and localization, translation rates and protein localization (Tian and Graber, 2012). Patterns of alternative polyadenylation are also regulated by differential binding of RNA binding proteins; CSTF2 and CFIm subunits of the main polyadenylation machinery have been shown to have effects on relative expression of alternatively polyadenylated isoforms (Zheng and Tian, 2014). Other RNA binding proteins such as HNRNPs H and I (Katz et al., 2010), as well as CPEB1 (Bava et al., 2013) have also been associated with alternative isoform choice. RBPs such as these may in the future form the basis of therapies to influence the 3′ end processing of alternatively polyadenylated transcripts as therapeutic agents.

### Therapeutic Modification of RNA Editing

RNA editing is a mechanism of generating further transcriptomic diversity and can impact the final sequence or structure of both encoded proteins and non-coding RNAs (ncRNAs) (Ganem and Lamm, 2017; Yablonovitch et al., 2017). RNA editing is an extremely common event, occurring in the many dynamically regulated mRNA transcripts and can comprise a variety of modifications, the most common of which is adenosine to inosine (A to I), which is eventually read as guanosine (Peng et al., 2012). RNA editing is especially prevalent in Small Interspersed Repetitive Elements (SINE) elements such as Alu, and also in transcripts in the brain (Jepson and Reenan, 2008; Osenberg et al., 2010). RNA editing events have been implicated in control of mRNA splicing and miRNA regulation (Farajollahi and Maas, 2010; Nishikura, 2010). RNA editing events are primarily mediated by a family of adenosine deaminases acting on RNA (ADARs), of which there are three major members; ADAR1, ADAR2, and ADAR3. The three ADARs have common functional domains, but differential structural features and some degree of site specificity (Nishikura, 2016). ADAR expression is itself regulated by transcription factors such as CREB and activated by kinases such as JNK1 (Peng et al., 2006; Yang et al., 2012). Dysfunction of ADAR1 is associated with diseases such as Aicardi-Goutières syndrome (Rice et al., 2012), with psychiatric disorders due to attenuated 5-HT_2C_R levels (Eran et al., 2013), and also with cancer (Ganem et al., 2017), whereas ADAR2 is linked to circadian rhythm and epilepsy (Gallo et al., 2017). Although less advanced than therapies targeting splicing defects, strategies to target ADARs to influence RNA editing are beginning to be evaluated for future clinical benefit. ADAR1 has been demonstrated to target let7, a miRNA involved in many processes including control of cell cycle (Roush and Slack, 2008). Over-expression of ADAR1 and subsequent down-regulation of Let7 has been shown to drive the self-renewal of leukemic stem cells in human blood, an observation that can be reversed by inhibition of ADAR1-mediated RNA editing (Zipeto et al., 2016). Very recently, techniques for directing ADARs to specific points of intervention have been developed. This system, named RESTORE, uses a plasmid-borne guide RNA coupled to an ADAR recruiting domain to deliver ADAR2 directly to the region of interest. This approach has been used to successfully edit phosphotyrosine residues in STAT1 with resultant changes to the activity of this signaling protein (Merkle et al., 2019). There are also newly emerging techniques based on modified Cas technologies utilizing catalytically inactive Cas13-ADAR2 fusion proteins to bring about RNA editing (Cox et al., 2017). These early observations suggest that in the future, targeting ADARs or other regulators of RNA editing may prove promising points of traction for neurodevelopmental disorders and for cancer.

### Modification of RNA Based Epitranscriptomics

Epitranscriptomic modification of DNA is well known, but it is now becoming increasingly evident that RNA is also epigenetically modified. RNA is subject to decoration with over 130 different modifications. Most of these map to very abundant RNAs such as rRNAs and tRNAs, but a subset are seen in mRNA, circRNA and lncRNA (Schaefer et al., 2017). The most common marks are *N*(6)-methyl-adenosine (m^6^A), 5-methylcytosine (m^5^C), 5-hydroxymethylcytosine (hm^5^C) and N1-methyladenosine (m^1^A), which have been shown to be widely present throughout the transcriptome by high throughput sequencing (Jung and Goldman, 2018). M^6^A is enriched in the last exon of genes and also occurs preferentially at 5′ untranslated regions (UTRs) (Ke et al., 2015; Meyer et al., 2015), whereas m^1^A is enriched in promoters and 5′ UTRs (Dominissini et al., 2016). m^5^C marks are often located at both 5′ and 3′ UTRs (Squires et al., 2012). RNA modifications can influence gene expression by a number of mechanisms, including influencing RNA structure, recruiting other regulatory proteins (e.g., splicing factors, RNA binding proteins involved in control of stability) or moderation of translation (Nachtergaele, 2017). RNA epitranscriptomic marks are added and removed by a series of writers (METTL3, METTL14, WTAP, KIAA1429, RBM15/15B, and METTL16) and erasers (FTO and ALKBH5) (Tong et al., 2018). Disruption of m^6^A disrupts RNA metabolism; m^6^A depleted transcripts have been reported to be unstable (Tang et al., 2018). Accordingly, mutations in the writer or eraser machinery have been associated with cancers such as hepatocellular carcinoma and acute myeloid leukemia (AML) (Vu et al., 2017; Chen et al., 2018), and with memory, fertility and metabolic phenotypes (Fischer et al., 2009; Zheng et al., 2013; Nainar et al., 2016). The RNA epigenomic writers and erasers are therefore promising future therapeutic targets. At present, the work in this area is mainly in cell and animal models. Silencing the METTL14 ‘writer’ led to restoration of differentiation of myeloid cells in AML and inhibited AML cell survival and proliferation (Weng et al., 2018). Similar strategies targeting ALKBH5 have showed promise as anti-tumor agents in glioblastoma stem cells (Schonberg et al., 2015). Studies have suggested that small molecule inhibitors of FTO may have potential utility as anticonvulsants in mouse models of epilepsy *in vivo*, by suppression of 2-oxoglutarate (2OG) through altering m^6^A levels (Zheng et al., 2014).

### Modulation of RNA Export

The activity of genes is also dependent on the correct positioning of mRNAs within the cell. Once processed, RNAs are usually exported through the nuclear membrane into the cytoplasm ready to be translated. This is not a passive process; it is orchestrated by a portfolio of RNA export proteins which escort the RNA molecule through the nuclear pore. Messenger RNAs are primarily transported by Nxf1 and Xpo1, whereas miRNAs are exported by Xpot and Xpo5. The transcription Export complex 1 (Trex1) facilitates binding of Nxf1 to the processed mRNA, and together with a collection of other proteins such as karyopherins or importins causes the processed mRNA to associate with and transit through the nuclear pore (Viphakone et al., 2012). The nuclear pore itself is composed of a collection of nucleoporins, and comprises a multi-subunit structure consisting of a nuclear ring, a central transport channel and a basket-like structure (Kabachinski and Schwartz, 2015). Small molecules can diffuse across this barrier, but larger ones such an mRNA cannot. Some of the specificity of transport is achieved by the interaction of the nuclear transport machinery with specific signal sequences in the mRNA itself (Lee et al., 2006; Hutten and Kehlenbach, 2007), whereas other mRNAs rely upon adaptor proteins (Huang et al., 2017). The expression and localization of nuclear transporters is altered in certain cancers (Zhou et al., 2013; Talati and Sweet, 2018), and have been linked with some neurodegenerative disorders (Grima et al., 2017) and comprises important components of inflammatory and apoptotic response (Aggarwal and Agrawal, 2014; Kopeina et al., 2018). Individual components of the nuclear export machinery are currently under investigation as therapeutics. One of the most promising, Selinexor, targets exportin 1 (Xpo1) and is currently in pre-clinical trials and has shown efficacy against acute myeloid leukemia and multiple myeloma (Kashyap et al., 2016; Mahipal and Malafa, 2016).

### Therapeutic Modulation of Non-coding RNA Regulators of Gene Expression

The repertoire of genes expressed by any given cell in any given circumstances is influenced by non-coding RNA (ncRNA) regulators of gene expression. These ncRNA genes do not encode proteins, but rather encode RNAs that contribute to the regulation of other RNAs. They are classified into 2 broad classes, short ncRNAs such as microRNAs (miRNAs) and longer ncRNAs such as long non-coding RNAS (lncRNAs) and circular RNAs (circRNAs).

#### Modulation of Small Non-coding RNAs

MicroRNAs (miRNAs) and siRNAs are short non-coding RNAs 20–25 bp in size. They interact with components of the RNA-induced silencing complex (RISC) to bring about translational blocking or RNA degradation. Each miRNA interacts with specific binding sites in the 3′ UTR of its target genes, which are 6–8 nt in length and are commonly found in the genome; each miRNA is thus capable of targeting hundreds of mRNA target genes simultaneously (Carthew and Sontheimer, 2009). Several classes of miRNAs have been associated with disease; these include the 17/92 cluster, the miR-24 cluster or miR-3676, all of which are associated with chronic lymphocytic leukemia (CLL) (Van Roosbroeck and Calin, 2016). Other examples include miR-21, miR-10b, miR-155, and Let-7a, which are associated with breast cancer (Khalighfard et al., 2018), and miR-192, miR200c and miR-17 which are associated with colon cancer (Ast et al., 2018). Similarly miR-33a and miR-33 have been associated with metabolic disease and atherosclerosis (Marquart et al., 2010; Rayner et al., 2011) and miR-155 has links with inflammatory diseases (Dorsett et al., 2008). The use of miRNAs as anti-tumor therapeutics is currently receiving much interest. Specific miRNAs can target the tumor suppressor machinery, and are commonly referred to as onco-miRs, or they may target the controls of cell cycle and act as tumor suppressors in their own right. The small size and relative stability of miRNAs and siRNAs, together with the observation that they are readily taken up in endosomes and microvesicles (Rani et al., 2017) renders them excellent candidates for therapeutic modulation or use as biomarkers of disease. This can take the form of antagomiRs that can target and silence endogenous miRNAs or chemically modified miRNA mimics that can increase regulation of their specific targets (Khvorova and Watts, 2017). To date, 20 clinical trials have been undertaken that exploit miRNA biology (Chakraborty et al., 2017), the first of which miravirsen, which is targeted to miR-122, is in phase II clinical trials for Hepatitis C (Lindow and Kauppinen, 2012). In August 2018, the first siRNA-related therapy, patisiran, was approved by the FDA for the treatment of peripheral nerve disease by targeting an abnormal form of the transthyretin (*TTR*) gene.

#### Modulation of LncRNAs

Long non-coding RNAs comprise a heterogeneous class of non-coding RNAs, which are longer than 200bp in length. They do not encode proteins, and originate from all most genomic regions. They can originate from the locus that they regulate, usually from the antisense strand, and regulate their target in *cis* (Natural antisense Transcripts (NATs), or they can map to entirely different genomic regions form their targets (introns, pseudogenes, and non-coding DNA) and cause regulation in *trans.* LncRNAs can also be associated with promoters, enhancers or other regulatory regions and do not have a homogeneous mode of action. They can activate or repress their targets and can work by a number of mechanisms. They are commonly involved in genomic imprinting; one of the first lncRNAs discovered, *XIST*, coordinates X chromosome inactivation (Brown et al., 1991). Other lncRNAs can act as guides. This class of lncRNA includes *ANRIL*, which directs the polycomb repressive complex to the site of action in the case of the *CDKN2A* and *CDKN2B* genes (Kotake et al., 2011) and the lncRNA *HOTAIR*, which has roles in colorectal cancer (Kogo et al., 2011). They can also act as scaffolds, directing the assembly of specific protein or RNA complexes to their sites of action. For example, one function of the lncRNA *NEAT1*, a multifunctional lncRNA with several roles in tumorigenesis (Ghaforui-Fard and Taheri, 2018) is to bring together the microRNA biogenesis machinery to enhance pri-miRNA processing (Jiang et al., 2017), and the lncRNA *LINP1*, which regulates the repair of DNA double strand breaks in breast cancer by acting as a scaffold for the ku80 and DNA-dependent protein kinase proteins (Zhang Y. et al., 2016). They can also repress expression by acting as decoys, co-regulators and inhibitors of RNA polymerase II. For example, the lncRNA *PANDA* acts by sequestering its transcription factor target NF-YA away from its site of action (Hung et al., 2011). They have roles in regulators of subcellular compartmentalization; the lncRNA *MALAT* is responsible for localizing splicing factors to the nuclear splicing speckles where they can be stored and regulated by phosphorylation (Bernard et al., 2010).

In accordance with their pivotal role in regulating gene expression, lncRNAs have been reported to be associated with several diseases such as cancer (Huarte, 2015; Parasramka et al., 2016; Peng et al., 2016), diabetes (Akerman et al., 2017; He et al., 2017; Leti and DiStefano, 2017), neurodegenerative disease (Riva et al., 2016) and cardiovascular disease (Hou et al., 2016; Haemmig et al., 2017; Gangwar et al., 2018). LncRNAs may represent promising therapeutic targets; they are responsive to small molecule therapeutics; a recent study documented 5916 lncRNAs that responded to 1262 small molecule drugs (Yang et al., 2017). Although progress toward the clinic has been slow, perhaps because of the diverse modes of actions of lncRNAs, there are some promising candidates. Several lncRNAs have been reported to be dysregulated in osteoarthritis (OA), including *HOTAIR, RP11-445H22.4, GAS5, PMS2L2, H19*, and *CTD-2574D22.4* (Xing et al., 2014). At the present time, the majority of studies have not progressed beyond cell or animal models, several potential future therapeutic candidates have emerged; the lncRNA *PCGEM1* was demonstrated to inhibit synoviocyte apoptosis on OA by moderation of its target miR-770 (Kang et al., 2016). Similarly, many lncRNAs have been identified as potential therapeutic targets in cardiovascular disease or cancer, including *GAS5, LIPCAR, SENCR, ANRIL, SMILR*, and *MALAT* (Gomes et al., 2017). ASP and siRNA approaches to therapeutically manipulate *MALAT* levels are in development in human cancer cells and in animal models (Arun et al., 2016). Targeting lncRNAs is subject to more difficulty than miRNAs, because of their larger size and the heterogeneity of their mode of action, which may explain why their evaluation is not as advanced as that of miRNAs. Nevertheless, they have significant potential as future therapeutic targets.

#### Modulation of CircRNAs

Circular RNAs (circRNAs) are a relatively newly discovered class of non-coding RNA regulators found in multiple species (Haque and Harries, 2017). They are formed from ‘backsplicing’ events of linear genes, and comprise circular molecules, which are therefore relatively immune to exonucleases (Cocquerelle et al., 1993; Schwanhausser et al., 2011; Jeck et al., 2013; Lan et al., 2016; Lasda and Parker, 2016). Like lncRNAs, circRNAs have been reported to influence gene expression by a variety of mechanisms including action as miRNA sponges or mRNA traps, as well as comprising modifiers of transcription. translation, or splicing (Haque and Harries, 2017). Circular RNAs have been suggested to have roles in many cellular processes, including embryonic development (Xia et al., 2016), metabolism (Xu et al., 2015), regulation of cell cycle (Zheng et al., 2016) and regulation of cellular stress (Burd et al., 2010). In accordance with this observation, dysregulated circRNA expression has been associated with multiple human diseases such as cancer (Yao et al., 2017), neurological disease (Khoutorsky et al., 2013), osteoarthritis (Liu et al., 2016), cardiovascular disease (Taibi et al., 2014; Wang et al., 2016), type 2 diabetes (Gu et al., 2017), pre-eclampsia (Zhang Y.G. et al., 2016) and impaired immune responses (Ng et al., 2016). Although the study of circRNAs is in its infancy compared with other ncRNAs, they too have potential as future therapeutic targets.

## Remaining Barriers and Future Prospects

**FIGURE 2:**
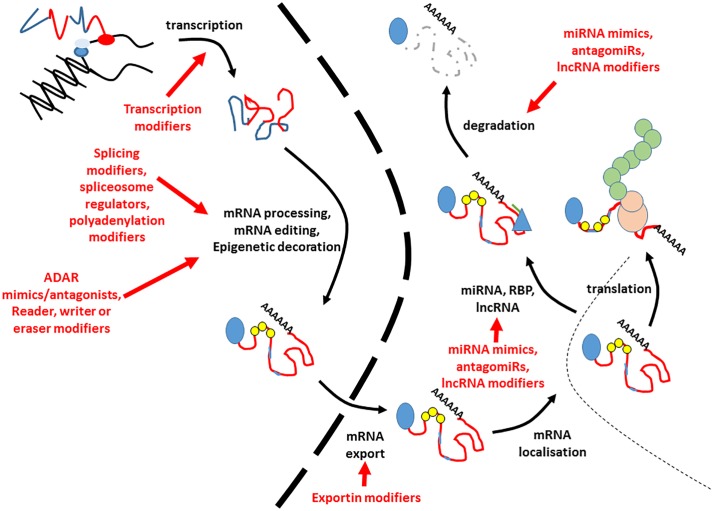
Potential points of intervention for RNA based therapies. This figure indicates the potential points at which interventions could be made to alter the amount or nature of expressed RNA. Blue lines in the transcript refer to introns and untranslated regions, whilst exons are indicted by red lines. The 5′ cap is indicated by a blue circle. Small yellow circles indicate epitranscriptomic decoration, whilst pale blue lines within the exons refer to RNA editing events. The nuclear envelope is indicated by a large dashed line. RNA binding proteins modifying stability are given by blue triangles, and miRNAs by green lines. The translating ribosome is indicated by beige circles. Nascent polypeptide is given by green interlocked circles. Each potential point of intervention is given by a red arrow. Degraded RNA is indicated by a gray dashed line.

This is an exciting time for RNA-based therapeutics, with several notable examples making it as far as license for clinical usage. Over the next decade, it is likely that there will be a large expansion in the breadth and scope of human disorders that can be treated using these, and similar approaches. Most developed at the present time, are interventions targeted at specific splice events and those involving small RNAs, but future work may harness the potential of targeting other parts of the RNA regulatory milieu (Figure 2).

Several barriers do, however, remain to the wide implementation of these opportunities which are focused mainly on delivery, specificity and duration of treatment. Firstly, delivery of specific molecules to their site of action may be challenging. For some applications, such as skin, which may be treated topically or lung, which may be treated via inhalation, therapeutic delivery of interventions may be easier. Delivery to internal organs such as brain, liver or pancreas will require different and systemic approaches. One reason why AONs, readthrough agents and small RNAs have been at the forefront of this emerging field is that their small size and relative stability means that they can be more easily introduced into cells. This may not be true of entities such as lncRNAs or large circRNAs, which may be large molecules with potentially challenging secondary or tertiary structure. Small molecules can readily be introduced into cells using lipid-mediated transfer agents, or endogenous structures such as endosomes or microvesicles, which could be harnessed to deliver cargoes. Secondly, there are questions of specificity. One feature of the therapies that are in clinic currently is their specificity to their sites of action. Gene expression and the regulation thereof is highly tissue specific, and genes may often be required to be expressed only at a specified time, or in response to specific circumstances. It may not be advantageous to produce changes in all tissues or at all times, and effects must of course be limited to their intended targets. Specificity of effect can be achieved by choosing targets that are only present at their sites of action, or by modifying delivery so that cargoes are only delivered to their intended place of action. For example, strategies are emerging now which allow selective delivery of senolytic cargoes to senescent cells only using galactosaccharide nanoparticles, which harness the observation that senescent cells harbor large quantities of lysosomal β-galactosidase (Munoz-Espin et al., 2018). Similarly, strategies could be developed that introduce therapeutic oligonucleotides under the control of gene regulatory elements expressed only in the intended target tissues. Lastly, one needs to consider the potential need for repeated treatments. The approaches discussed here differ from emerging “gene editing” technologies such as CRISPR, in that they are not transmitted to future generations, and may require repeated treatments. This can be considered both a caveat and an advantage. The need for repeated treatments may be burdensome for patients, but in reality, the vast majority of currently available treatments for human disorders fall into this category. Conversely, the need to deliver repeated doses introduces a degree of flexibility, and allows treatments to be quickly discontinued or changed if adverse effects occur. We are at a time of huge advances in our understanding of how our genome is curated and regulated and how our genes are expressed.

The multifactorial control of gene expression, and the complexity of this progress offers multiple points of potential intervention for therapeutic benefit. Over the coming decades, there is likely to be a huge increase in the number of therapies for human diseases that target not the genes themselves, but the expression and regulation of those genes. We are at the dawning of the era of genomic medicine, and the future looks bright.

## Author Contributions

LH planned and wrote the manuscript.

## Conflict of Interest Statement

The author declares that the research was conducted in the absence of any commercial or financial relationships that could be construed as a potential conflict of interest.

## References

[B1] Aartsma-Rus A. (2010). Antisense-mediated modulation of splicing: therapeutic implications for Duchenne muscular dystrophy. *RNA Biol.* 7 453–461. 20523110 10.4161/rna.7.4.12264

[B2] Aggarwal A. Agrawal D. K. (2014). Importins, and exportins regulating allergic immune responses. *Med. Inflamm.* 2014:476357. 10.1155/2014/476357 24733961 PMC3964845

[B3] Akerman I. Tu Z. Beucher A. Rolando D. M. Y. Sauty-Colace C. Benazra M. (2017). Human pancreatic beta cell lncRNAs control cell-specific regulatory networks. *Cell Metab.* 25 400–411. 10.1016/j.cmet.2016.11.016 28041957 PMC5300904

[B4] Araki S. Dairiki R. Nakayama Y. Murai A. Miyashita R. Iwatani M. (2015). Inhibitors of CLK protein kinases suppress cell growth and induce apoptosis by modulating pre-mRNA splicing. *PLoS One* 10:e0116929. 10.1371/journal.pone.0116929 25581376 PMC4291223

[B5] Arun G. Diermeier S. Akerman M. Chang K. C. Wilkinson J. E. Hearn S. (2016). Differentiation of mammary tumors and reduction in metastasis upon Malat1 lncRNA loss. *Genes Dev.* 30 34–51. 10.1101/gad.270959.115 26701265 PMC4701977

[B6] Ast V. Kordass T. Oswald M. Kolte A. Eisel D. Osen W. (2018). MiR-192, miR-200c and miR-17 are fibroblast-mediated inhibitors of colorectal cancer invasion. *Oncotarget* 9 35559–35580. 10.18632/oncotarget.26263 30473751 PMC6238973

[B7] Bates D. O. Morris J. C. Oltean S. Donaldson L. F. (2017). Pharmacology of modulators of alternative splicing. *Pharmacol. Rev.* 69 63–79. 10.1124/pr.115.011239 28034912 PMC5226212

[B8] Bava F. A. Eliscovich C. Ferreira P. G. Minana B. Ben-Dov C. Guigo R. (2013). CPEB1 coordinates alternative 3′-UTR formation with translational regulation. *Nature* 495 121–125. 10.1038/nature11901 23434754

[B9] Beaulieu C. L. Majewski J. Schwartzentruber J. Samuels M. E. Fernandez B. A. Bernier F. P. (2014). FORGE Canada Consortium: outcomes of a 2-year national rare-disease gene-discovery project. *Am. J. Hum. Genet.* 94 809–817. 10.1016/j.ajhg.2014.05.003 24906018 PMC4121481

[B10] Bentley D. (2002). The mRNA assembly line: transcription and processing machines in the same factory. *Curr. Opin. Cell Biol.* 14 336–342. 12067656 10.1016/s0955-0674(02)00333-2

[B11] Bernard D. Prasanth K. V. Tripathi V. Colasse S. Nakamura T. Xuan Z. (2010). A long nuclear-retained non-coding RNA regulates synaptogenesis by modulating gene expression. *EMBO J.* 29 3082–3093. 10.1038/emboj.2010.199 20729808 PMC2944070

[B12] Berson A. Barbash S. Shaltiel G. Goll Y. Hanin G. Greenberg D. S. (2012). Cholinergic-associated loss of hnRNP-A/B in Alzheimer’s disease impairs cortical splicing and cognitive function in mice. *EMBO Mol. Med.* 4 730–742. 10.1002/emmm.201100995 22628224 PMC3494073

[B13] Beyer A. L. Osheim Y. N. (1988). Splice site selection, rate of splicing, and alternative splicing on nascent transcripts. *Genes Dev.* 2 754–765. 3138163 10.1101/gad.2.6.754

[B14] Blencowe B. J. (2000). Exonic splicing enhancers: mechanism of action, diversity and role in human genetic diseases. *Trends Biochem. Sci.* 25 106–110. 10694877 10.1016/s0968-0004(00)01549-8

[B15] Borbolis F. Syntichaki P. (2015). Cytoplasmic mRNA turnover and ageing. *Mech. Ageing Dev.* 152 32–42. 26432921 10.1016/j.mad.2015.09.006 PMC4710634

[B16] Brown C. J. Lafreniere R. G. Powers V. E. Sebastio G. Ballabio A. Pettigrew A. L. (1991). Localization of the X inactivation centre on the human X chromosome in Xq13. *Nature* 349 82–84. 1985270 10.1038/349082a0

[B17] Burd C. E. Jeck W. R. Liu Y. Sanoff H. K. Wang Z. Sharpless N. E. (2010). Expression of linear and novel circular forms of an INK4/ARF-associated non-coding RNA correlates with atherosclerosis risk. *PLoS Genet.* 6:e1001233. 10.1371/journal.pgen.1001233 21151960 PMC2996334

[B18] Cartegni L. Chew S. L. Krainer A. R. (2002). Listening to silence and understanding nonsense: exonic mutations that affect splicing. *Nat. Rev. Genet.* 3 285–298. 11967553 10.1038/nrg775

[B19] Carthew R. W. Sontheimer E. J. (2009). Origins and Mechanisms of miRNAs and siRNAs. *Cell* 136 642–655. 10.1016/j.cell.2009.01.035 19239886 PMC2675692

[B20] Chakraborty C. Sharma A. R. Sharma G. Doss C. G. P. Lee S. S. (2017). Therapeutic miRNA and siRNA: moving from bench to clinic as next generation medicine. *Mol. Ther. Nucleic Acids* 8 132–143. 10.1016/j.omtn.2017.06.005 28918016 PMC5496203

[B21] Chen L. (2013). Characterization and comparison of human nuclear and cytosolic editomes. *Proc. Natl. Acad. Sci. U.S.A.* 110 E2741–E2747. 10.1073/pnas.1218884110 23818636 PMC3718162

[B22] Chen M. Wei L. Law C. T. Tsang F. H. Shen J. Cheng C. L. (2018). RNA N6-methyladenosine methyltransferase-like 3 promotes liver cancer progression through YTHDF2-dependent posttranscriptional silencing of SOCS2. *Hepatology* 67 2254–2270. 10.1002/hep.29683 29171881

[B23] Chen W. Jia Q. Song Y. Fu H. Wei G. Ni T. (2017). Alternative polyadenylation: methods, findings, and impacts. *Genomics Proteomics Bioinformatics* 15 287–300. 10.1016/j.gpb.2017.06.001 29031844 PMC5673674

[B24] Cnop M. Abdulkarim B. Bottu G. Cunha D. A. Igoillo-Esteve M. Masini M. (2014). RNA sequencing identifies dysregulation of the human pancreatic islet transcriptome by the saturated fatty acid palmitate. *Diabetes* 63 1978–1993. 10.2337/db13-1383 24379348

[B25] Cocquerelle C. Mascrez B. Hetuin D. Bailleul B. (1993). Mis-splicing yields circular RNA molecules. *FASEB J.* 7 155–160. 7678559 10.1096/fasebj.7.1.7678559

[B26] Cox D. B. T. Gootenberg J. S. Abudayyeh O. O. Franklin B. Kellner M. J. Joung J. (2017). RNA editing with CRISPR-Cas13. *Science* 358 1019–1027. 10.1126/science.aaq0180 29070703 PMC5793859

[B27] Dominissini D. Nachtergaele S. Moshitch-Moshkovitz S. Peer E. Kol N. Ben-Haim M. S. (2016). The dynamic N(1)-methyladenosine methylome in eukaryotic messenger RNA. *Nature* 530 441–446. 10.1038/nature16998 26863196 PMC4842015

[B28] Dorsett Y. McBride K. M. Jankovic M. Gazumyan A. Thai T. H. Robbiani D. F. (2008). MicroRNA-155 suppresses activation-induced cytidine deaminase-mediated Myc-Igh translocation. *Immunity* 28 630–638. 10.1016/j.immuni.2008.04.002 18455451 PMC2713656

[B29] Edwards A. Y. Elgart A. Farrell C. Barnett-Griness O. Rabinovich-Guilatt L. Spiegelstein O. (2017). A population pharmacokinetic meta-analysis of custirsen, an antisense oligonucleotide, in oncology patients and healthy subjects. *Br. J. Clin. Pharmacol.* 83 1932–1943. 10.1111/bcp.13287 28294391 PMC5555861

[B30] Elkon R. Ugalde A. P. Agami R. (2013). Alternative cleavage and polyadenylation: extent, regulation and function. *Nat. Rev. Genet.* 14 496–506. 10.1038/nrg3482 23774734

[B31] Eran A. Li J. B. Vatalaro K. McCarthy J. Rahimov F. Collins C. (2013). Comparative RNA editing in autistic and neurotypical cerebella. *Mol. Psychiatry* 18 1041–1048. 22869036 10.1038/mp.2012.118 PMC3494744

[B32] Farajollahi S. Maas S. (2010). Molecular diversity through RNA editing: a balancing act. *Trends Genet.* 26 221–230. 10.1016/j.tig.2010.02.001 20395010 PMC2865426

[B33] Figlin R. A. (2015). Personalized immunotherapy ( AGS-003 ) when combined with sunitinib for the treatment of metastatic renal cell carcinoma. *Expert Opin. Biol. Ther.* 15 1241–1248. 10.1517/14712598.2015.1063610 26125651

[B34] Fischer J. Koch L. Emmerling C. Vierkotten J. Peters T. Bruning J. C. (2009). Inactivation of the Fto gene protects from obesity. *Nature* 458 894–898. 10.1038/nature07848 19234441

[B35] Fukao A. Aoyama T. Fujiwara T. (2015). The molecular mechanism of translational control via the communication between the microRNA pathway and RNA-binding proteins. *RNA Biol.* 12 922–926. 10.1080/15476286.2015.1073436 26274611 PMC4615170

[B36] Fukao A. Fujiwara T. (2017). The coupled and uncoupled mechanisms by which trans-acting factors regulate mRNA stability and translation. *J. Biochem.* 161 309–314. 10.1093/jb/mvw086 28039391

[B37] Gallo A. Vukic D. Michalik D. O’Connell M. A. Keegan L. P. (2017). ADAR RNA editing in human disease; more to it than meets the I. *Hum. Genet.* 136 1265–1278. 10.1007/s00439-017-1837-0 28913566

[B38] Ganem N. S. Ben-Asher N. Lamm A. T. (2017). In cancer, A-to-I RNA editing can be the driver, the passenger, or the mechanic. *Drug Resist. Updat.* 32 16–22. 10.1016/j.drup.2017.09.001 29145975

[B39] Ganem N. S. Lamm A. T. (2017). A-to-I RNA editing - thinking beyond the single nucleotide. *RNA Biol.* 14 1690–1694. 10.1080/15476286.2017.1364830 28820319 PMC5731795

[B40] Gangwar R. S. Rajagopalan S. Natarajan R. Deiuliis J. A. (2018). Noncoding RNAs in cardiovascular disease: pathological relevance and emerging role as biomarkers and therapeutics. *Am. J. Hypertens.* 31 150–165. 10.1093/ajh/hpx197 29186297 PMC5861558

[B41] Ghaforui-Fard S. Taheri M. (2018). Nuclear Enriched Abundant Transcript 1 (NEAT1): a long non-coding RNA with diverse functions in tumorigenesis. *Biomed. Pharmacother.* 111 51–59. 10.1016/j.biopha.2018.12.070 30576934

[B42] Goemans N. M. Tulinius M. van den Akker J. T. Burm B. E. Ekhart P. F. Heuvelmans N. (2011). Systemic administration of PRO051 in Duchenne’s muscular dystrophy. *N. Engl. J. Med.* 364 1513–1522. 10.1056/NEJMoa1011367 21428760

[B43] Gomes C. P. C. Spencer H. Ford K. L. Michel L. Y. M. Baker A. H. Emanueli C. (2017). The function and therapeutic potential of long non-coding RNAs in cardiovascular development and disease. *Mol. Ther. Nucleic Acids* 8 494–507. 28918050 10.1016/j.omtn.2017.07.014 PMC5565632

[B44] Gorgoni B. Marshall E. McFarland M. R. Romano M. C. Stansfield I. (2014). Controlling translation elongation efficiency: tRNA regulation of ribosome flux on the mRNA. *Biochem. Soc. Trans.* 42 160–165. 10.1042/BST20130132 24450645

[B45] Grima J. C. Daigle J. G. Arbez N. Cunningham K. C. Zhang K. Ochaba J. (2017). Mutant huntingtin disrupts the nuclear pore complex. *Neuron* 94 93–107.e6. 10.1016/j.neuron.2017.03.023 28384479 PMC5595097

[B46] Gu Y. Ke G. Wang L. Zhou E. Zhu K. Wei Y. (2017). Altered expression profile of circular RNAs in the serum of patients with diabetic retinopathy revealed by microarray. *Ophthalm. Res.* 58 176–184. 10.1159/000479156 28817829

[B47] Haemmig S. Simion V. Yang D. Deng Y. Feinberg M. W. (2017). Long noncoding RNAs in cardiovascular disease, diagnosis, and therapy. *Curr. Opin. Cardiol.* 32 776–783. 10.1097/HCO.0000000000000454 28786864 PMC5892448

[B48] Haque S. Harries L. W. (2017). Circular RNAs (circRNAs) in health and disease. *Genes* 8 E353. 10.3390/genes8120353 PMC5748671 29182528

[B49] Harigaya Y. Parker R. (2010). No-go decay: a quality control mechanism for RNA in translation. *Wiley Interdiscip. Rev. RNA* 1 132–141. 10.1002/wrna.17 21956910

[B50] He X. Ou C. Xiao Y. Han Q. Li H. Zhou S. (2017). LncRNAs: key players and novel insights into diabetes mellitus. *Oncotarget* 8 71325–71341. 10.18632/oncotarget.19921 29050364 PMC5642639

[B51] Helm M. Motorin Y. (2017). Detecting RNA modifications in the epitranscriptome: predict and validate. *Nat. Rev. Genet.* 18 275–291. 10.1038/nrg.2016.169 28216634

[B52] Holly A. C. Melzer D. Pilling L. C. Fellows A. C. Tanaka T. Ferrucci L. (2013). Changes in splicing factor expression are associated with advancing age in man. *Mech. Ageing Dev.* 134 356–366. 10.1016/j.mad.2013.05.006 23747814 PMC5863542

[B53] Hou J. Zhou C. Long H. Zheng S. Guo T. Wu Q. (2016). Long noncoding RNAs: novel molecules in cardiovascular biology, disease and regeneration. *Exp. Mol. Pathol.* 100 493–501. 10.1016/j.yexmp.2016.05.006 27180105

[B54] Huang J. H. Ku W. C. Chen Y. C. Chang Y. L. Chu C. Y. (2017). Dual mechanisms regulate the nucleocytoplasmic localization of human DDX6. *Sci. Rep.* 7:42853. 10.1038/srep42853 28216671 PMC5316971

[B55] Huarte M. (2015). The emerging role of lncRNAs in cancer. *Nat. Med.* 21 1253–1261. 26540387 10.1038/nm.3981

[B56] Huisman C. van der Wijst M. G. Falahi F. Overkamp J. Karsten G. Terpstra M. M. (2015). Prolonged re-expression of the hypermethylated gene EPB41L3 using artificial transcription factors and epigenetic drugs. *Epigenetics* 10 384–396. 10.1080/15592294.2015.1034415 25830725 PMC4622424

[B57] Hung T. Wang Y. Lin M. F. Koegel A. K. Kotake Y. Grant G. D. (2011). Extensive and coordinated transcription of noncoding RNAs within cell-cycle promoters. *Nat. Genet.* 43 621–629. 10.1038/ng.848 21642992 PMC3652667

[B58] Hutten S. Kehlenbach R. H. (2007). CRM1-mediated nuclear export: to the pore and beyond. *Trends Cell Biol.* 17 193–201. 17317185 10.1016/j.tcb.2007.02.003

[B59] Iadevaia V. Gerber A. P. (2015). Combinatorial control of mRNA Fates by RNA-binding proteins and non-coding RNAs. *Biomolecules* 5 2207–2222. 10.3390/biom5042207 26404389 PMC4693235

[B60] Jeck W. R. Sorrentino J. A. Wang K. Slevin M. K. Burd C. E. Liu J. (2013). Circular RNAs are abundant, conserved, and associated with ALU repeats. *RNA* 19 141–157. 10.1261/rna.035667.112 23249747 PMC3543092

[B61] Jepson J. E. Reenan R. A. (2008). RNA editing in regulating gene expression in the brain. *Biochim. Biophys. Acta* 1779 459–470. 18086576 10.1016/j.bbagrm.2007.11.009

[B62] Jiang L. Shao C. Wu Q. J. Chen G. Zhou J. Yang B. (2017). NEAT1 scaffolds RNA-binding proteins and the microprocessor to globally enhance pri-miRNA processing. *Nat. Struct. Mol. Biol.* 24 816–824. 10.1038/nsmb.3455 28846091 PMC5766049

[B63] Jung Y. Goldman D. (2018). Role of RNA modifications in brain and behavior. *Genes Brain Behav.* 17:e12444. 10.1111/gbb.12444 29244246 PMC6233296

[B64] Kabachinski G. Schwartz T. U. (2015). The nuclear pore complex–structure and function at a glance. *J. Cell Sci.* 128 423–429. 26046137 10.1242/jcs.083246 PMC4311126

[B65] Kang Y. Song J. Kim D. Ahn C. Park S. Chun C. H. (2016). PCGEM1 stimulates proliferation of osteoarthritic synoviocytes by acting as a sponge for miR-770. *J. Orthop. Res.* 34 412–418. 10.1002/jor.23046 26340084

[B66] Kashyap T. Argueta C. Aboukameel A. Unger T. J. Klebanov B. Mohammad R. M. (2016). Selinexor, a selective inhibitor of nuclear export (SINE) compound, acts through NF-kappaB deactivation and combines with proteasome inhibitors to synergistically induce tumor cell death. *Oncotarget* 7 78883–78895. 10.18632/oncotarget.12428 27713151 PMC5346685

[B67] Katz Y. Wang E. T. Airoldi E. M. Burge C. B. (2010). Analysis and design of RNA sequencing experiments for identifying isoform regulation. *Nat. Methods* 7 1009–1015. 10.1038/nmeth.1528 21057496 PMC3037023

[B68] Ke S. Alemu E. A. Mertens C. Gantman E. C. Fak J. J. Mele A. (2015). A majority of m6A residues are in the last exons, allowing the potential for 3′. UTR regulation. *Genes Dev.* 29 2037–2053. 10.1101/gad.269415.115 26404942 PMC4604345

[B69] Khalighfard S. Alizadeh A. M. Irani S. Omranipour R. (2018). Plasma miR-21, miR-155, miR-10b, and Let-7a as the potential biomarkers for the monitoring of breast cancer patients. *Sci. Rep.* 8:17981. 10.1038/s41598-018-36321-3 30568292 PMC6299272

[B70] Khoutorsky A. Yanagiya A. Gkogkas C. G. Fabian M. R. Prager-Khoutorsky M. Cao R. (2013). Control of synaptic plasticity and memory via suppression of poly(A)-binding protein. *Neuron* 78 298–311. 10.1016/j.neuron.2013.02.025 23622065

[B71] Khvorova A. Watts J. K. (2017). The chemical evolution of oligonucleotide therapies of clinical utility. *Nat. Biotechnol.* 35 238–248. 10.1038/nbt.3765 28244990 PMC5517098

[B72] Klauer A. A. van Hoof A. (2012). Degradation of mRNAs that lack a stop codon: a decade of nonstop progress. *Wiley Interdiscip. Rev. RNA* 3 649–660. 10.1002/wrna.1124 22740367 PMC3638749

[B73] Kogo R. Shimamura T. Mimori K. Kawahara K. Imoto S. Sudo T. (2011). Long noncoding RNA HOTAIR regulates polycomb-dependent chromatin modification and is associated with poor prognosis in colorectal cancers. *Cancer Res.* 71 6320–6326. 10.1158/0008-5472.CAN-11-1021 21862635

[B74] Kopeina G. S. Prokhorova E. A. Lavrik I. N. Zhivotovsky B. (2018). Alterations in the nucleocytoplasmic transport in apoptosis: caspases lead the way. *Cell Prolif.* 51:e12467. 10.1111/cpr.12467 29947118 PMC6528946

[B75] Kotake Y. Nakagawa T. Kitagawa K. Suzuki S. Liu N. Kitagawa M. (2011). Long non-coding RNA ANRIL is required for the PRC2 recruitment to and silencing of p15(INK4B) tumor suppressor gene. *Oncogene* 30 1956–1962. 10.1038/onc.2010.568 21151178 PMC3230933

[B76] Lan P. H. Liu Z. H. Pei Y. J. Wu Z. G. Yu Y. Yang Y. F. (2016). Landscape of RNAs in human lumbar disc degeneration. *Oncotarget* 7 63166–63176. 10.18632/oncotarget.11334 27542248 PMC5325354

[B77] Lasda E. Parker R. (2016). Circular RNAs Co-precipitate with extracellular vesicles: a possible mechanism for circRNA clearance. *PLoS One* 11:e0148407. 10.1371/journal.pone.0148407 26848835 PMC4743949

[B78] Latorre E. Birar V. C. Sheerin A. N. Jeynes J. C. C. Hooper A. Dawe H. R. (2017). Small molecule modulation of splicing factor expression is associated with rescue from cellular senescence. *BMC Cell Biol.* 18:31. 10.1186/s12860-017-0147-7 29041897 PMC5645932

[B79] Latorre E. Ostler E. O. Faragher R. G. A. Harries L. W. (2018). FOXO1 and ETV6 genes may represent novel regulators of splicing factor expression in cellular senescence. *FASEB J.* 33 1086–1097. 10.1096/fj.201801154R 30088951

[B80] Lee B. J. Cansizoglu A. E. Suel K. E. Louis T. H. Zhang Z. Chook Y. M. (2006). Rules for nuclear localization sequence recognition by karyopherin beta 2. *Cell* 126 543–558. 16901787 10.1016/j.cell.2006.05.049 PMC3442361

[B81] Lefebvre S. Burglen L. Reboullet S. Clermont O. Burlet P. Viollet L. (1995). Identification and characterization of a spinal muscular atrophy-determining gene. *Cell* 80 155–165. 7813012 10.1016/0092-8674(95)90460-3

[B82] Lejeune F. (2017). Nonsense-mediated mRNA decay at the crossroads of many cellular pathways. *BMB Rep.* 50 175–185. 28115040 10.5483/BMBRep.2017.50.4.015 PMC5437961

[B83] Leti F. DiStefano J. K. (2017). Long Noncoding RNAs as Diagnostic and Therapeutic Targets in Type 2 Diabetes and Related Complications. *Genes* 8 E207. 10.3390/genes8080207 28829354 PMC5575670

[B84] Lindow M. Kauppinen S. (2012). Discovering the first microRNA-targeted drug. *J. Cell Biol.* 199 407–412. 10.1083/jcb.201208082 23109665 PMC3483128

[B85] Liu Q. Zhang X. Hu X. Dai L. Fu X. Zhang J. (2016). Circular RNA related to the chondrocyte ECM regulates MMP13 expression by functioning as a MiR-136 ‘Sponge’ in human cartilage degradation. *Sci. Rep.* 6:22572. 10.1038/srep22572 26931159 PMC4773870

[B86] Lorson C. L. Hahnen E. Androphy E. J. Wirth B. (1999). A single nucleotide in the SMN gene regulates splicing and is responsible for spinal muscular atrophy. *Proc. Natl. Acad. Sci. U.S.A.* 96 6307–6311. 10339583 10.1073/pnas.96.11.6307 PMC26877

[B87] Love J. E. Hayden E. J. Rohn T. T. (2015). Alternative splicing in Alzheimer’s disease. *J. Parkinsons Dis. Alzheimers Dis.* 2:6. 10.13188/2376-922X.1000010 PMC4772657 26942228

[B88] Lu Z. X. Huang Q. Park J. W. Shen S. Lin L. Tokheim C. J. (2015). Transcriptome-wide landscape of pre-mRNA alternative splicing associated with metastatic colonization. *Mol. Cancer Res.* 13 305–318. 10.1158/1541-7786.MCR-14-0366 25274489 PMC4336826

[B89] Mahipal A. Malafa M. (2016). Importins, and exportins as therapeutic targets in cancer. *Pharmacol. Ther.* 164 135–143. 27113410 10.1016/j.pharmthera.2016.03.020

[B90] Makarov E. M. Makarova O. V. Urlaub H. Gentzel M. Will C. L. Wilm M. (2002). Small nuclear ribonucleoprotein remodeling during catalytic activation of the spliceosome. *Science* 298 2205–2208. 12411573 10.1126/science.1077783

[B91] Manolio T. A. Brooks L. D. Collins F. S. (2008). A HapMap harvest of insights into the genetics of common disease. *J. Clin. Investig.* 118 1590–1605. 10.1172/JCI34772 18451988 PMC2336881

[B92] Marquart T. J. Allen R. M. Ory D. S. Baldan A. (2010). miR-33 links SREBP-2 induction to repression of sterol transporters. *Proc. Natl. Acad. Sci. U.S.A.* 107 12228–12232. 10.1073/pnas.1005191107 20566875 PMC2901433

[B93] Mavrou A. Brakspear K. Hamdollah-Zadeh M. Damodaran G. Babaei-Jadidi R. Oxley J. (2015). Serine-arginine protein kinase 1 (SRPK1) inhibition as a potential novel targeted therapeutic strategy in prostate cancer. *Oncogene* 34 4311–4319. 10.1038/onc.2014.360 25381816 PMC4351909

[B94] Mendell J. R. Shilling C. Leslie N. D. Flanigan K. M. al-Dahhak R. Gastier-Foster J. (2012). Evidence-based path to newborn screening for duchenne muscular dystrophy. *Ann. Neurol.* 71 304–313. 10.1002/ana.23528 22451200

[B95] Merkle T. Merz S. Reautschnig P. Blaha A. Li Q. Vogel P. (2019). Precise RNA editing by recruiting endogenous ADARs with antisense oligonucleotides. *Nat. Biotechnol.* 37 133–138. 10.1038/s41587-019-0013-6 30692694

[B96] Meyer K. D. Patil D. P. Zhou J. Zinoviev A. Skabkin M. A. Elemento O. (2015). 5′ UTR m(6)A promotes cap-independent translation. *Cell* 163 999–1010. 10.1016/j.cell.2015.10.012 26593424 PMC4695625

[B97] Monaco A. P. Bertelson C. J. Liechti-Gallati S. Moser H. Kunkel L. M. (1988). An explanation for the phenotypic differences between patients bearing partial deletions of the DMD locus. *Genomics* 2 90–95. 3384440 10.1016/0888-7543(88)90113-9

[B98] Munoz-Espin D. Rovira M. Galiana I. Gimenez C. Lozano-Torres B. Paez-Ribes M. (2018). A versatile drug delivery system targeting senescent cells. *EMBO Mol. Med.* 10:e9355. 10.15252/emmm.201809355 30012580 PMC6127887

[B99] Nachtergaele S. (2017). He C. The emerging biology of RNA post-transcriptional modifications. *RNA Biol.* 14 156–163. 10.1080/15476286.2016.1267096 27937535 PMC5324755

[B100] Nainar S. Marshall P. R. Tyler C. R. Spitale R. C. Bredy T. W. (2016). Evolving insights into RNA modifications and their functional diversity in the brain. *Nat. Neurosci.* 19 1292–1298. 10.1038/nn.4378 27669990 PMC5068363

[B101] Nakajima H. Hori Y. Terano H. Okuhara M. Manda T. Matsumoto S. (1996). New antitumor substances, FR901463, FR901464 and FR901465. II. Activities against experimental tumors in mice and mechanism of action. *J. Antibiot.* 49 1204–1211. 9031665 10.7164/antibiotics.49.1204

[B102] Namgoong J. H. Bertoni C. (2016). Clinical potential of ataluren in the treatment of Duchenne muscular dystrophy. *Degener. Neurol. Neuromuscul. Dis.* 6 37–48. 10.2147/DNND.S71808 30050367 PMC6053089

[B103] Ng W. L. Marinov G. K. Liau E. S. Lam Y. L. Lim Y. Y. Ea C. K. (2016). Inducible RasGEF1B circular RNA is a positive regulator of ICAM-1 in the TLR4/LPS pathway. *RNA Biol.* 13 861–871. 10.1080/15476286.2016.1207036 27362560 PMC5014010

[B104] Niks E. H. Aartsma-Rus A. (2017). Exon skipping: a first in class strategy for Duchenne muscular dystrophy. *Expert Opin. Biol. Ther.* 17 225–236. 10.1080/14712598.2017.1271872 27936976

[B105] Nishikura K. (2010). Functions and regulation of RNA editing by ADAR deaminases. *Annu. Rev. Biochem.* 79 321–349. 10.1146/annurev-biochem-060208-105251 20192758 PMC2953425

[B106] Nishikura K. (2016). A-to-I editing of coding and non-coding RNAs by ADARs. *Nat. Rev. Mol. Cell Biol.* 17 83–96. 10.1038/nrm.2015.4 26648264 PMC4824625

[B107] Osenberg S. Paz Yaacov N. Safran M. Moshkovitz S. Shtrichman R. Sherf O. (2010). Alu sequences in undifferentiated human embryonic stem cells display high levels of A-to-I RNA editing. *PLoS One* 5:e11173. 10.1371/journal.pone.0011173 20574523 PMC2888580

[B108] Pan Q. Shai O. Lee L. J. Frey B. J. Blencowe B. J. (2008). Deep surveying of alternative splicing complexity in the human transcriptome by high-throughput sequencing. *Nat. Genet.* 40 1413–1415. 10.1038/ng.259 18978789

[B109] Parasramka M. A. Maji S. Matsuda A. Yan I. K. Patel T. (2016). Long non-coding RNAs as novel targets for therapy in hepatocellular carcinoma. *Pharmacol. Ther.* 161 67–78. 27013343 10.1016/j.pharmthera.2016.03.004 PMC4851900

[B110] Parente V. Corti S. (2018). Advances in spinal muscular atrophy therapeutics. *Ther. Adv. Neurol. Disord.* 11:1756285618754501. 10.1177/1756285618754501 29434670 PMC5802612

[B111] Peng L. Yuan X. Jiang B. Tang Z. (2016). Li GC. LncRNAs: key players and novel insights into cervical cancer. *Tumour Biol.* 37 2779–2788. 10.1007/s13277-015-4663-9 26715267

[B112] Peng P. L. Zhong X. Tu W. Soundarapandian M. M. Molner P. Zhu D. (2006). ADAR2-dependent RNA editing of AMPA receptor subunit GluR2 determines vulnerability of neurons in forebrain ischemia. *Neuron* 49 719–733. 16504947 10.1016/j.neuron.2006.01.025

[B113] Peng Z. Cheng Y. Tan B. C. Kang L. Tian Z. Zhu Y. (2012). Comprehensive analysis of RNA-Seq data reveals extensive RNA editing in a human transcriptome. *Nat. Biotechnol.* 30 253–260. 10.1038/nbt.2122 22327324

[B114] Rani A. O’Shea A. Ianov L. Cohen R. A. Woods A. J. Foster T. C. (2017). miRNA in circulating microvesicles as biomarkers for age-related cognitive decline. *Front. Aging Neurosci.* 9:323. 10.3389/fnagi.2017.00323 29046635 PMC5632661

[B115] Rayner K. J. Sheedy F. J. Esau C. C. Hussain F. N. Temel R. E. Parathath S. (2011). Antagonism of miR-33 in mice promotes reverse cholesterol transport and regression of atherosclerosis. *J. Clin. Investig.* 121 2921–2931. 10.1172/JCI57275 21646721 PMC3223840

[B116] Rice G. I. Kasher P. R. Forte G. M. Mannion N. M. Greenwood S. M. Szynkiewicz M. (2012). Mutations in ADAR1 cause aicardi-goutieres syndrome associated with a type I interferon signature. *Nat. Genet.* 44 1243–1248. 10.1038/ng.2414 23001123 PMC4154508

[B117] Riva P. Ratti A. Venturin M. (2016). The long non-coding RNAs in neurodegenerative diseases: novel mechanisms of pathogenesis. *Curr. Alzheimer Res.* 13 1219–1231. 27338628 10.2174/1567205013666160622112234

[B118] Rizk M. Tuzmen S. (2017). Update on the clinical utility of an RNA interference-based treatment: focus on Patisiran. *Pharmgenomics Pers. Med.* 10 267–278. 10.2147/PGPM.S87945 29184431 PMC5689029

[B119] Roush S. Slack F. J. (2008). The let-7 family of microRNAs. *Trends Cell Biol.* 18 505–516. 18774294 10.1016/j.tcb.2008.07.007

[B120] Sandberg R. Neilson J. R. Sarma A. Sharp P. A. Burge C. B. (2008). Proliferating cells express mRNAs with shortened 3’ untranslated regions and fewer microRNA target sites. *Science* 320 1643–1647. 10.1126/science.1155390 18566288 PMC2587246

[B121] Schaefer M. Kapoor U. Jantsch M. F. (2017). Understanding RNA modifications: the promises and technological bottlenecks of the ‘epitranscriptome’. *Open Biol.* 7:170077. 10.1098/rsob.170077 28566301 PMC5451548

[B122] Schonberg D. L. Miller T. E. Wu Q. Flavahan W. A. Das N. K. Hale J. S. (2015). Preferential iron trafficking characterizes glioblastoma stem-like cells. *Cancer Cell* 28 441–455. 10.1016/j.ccell.2015.09.002 26461092 PMC4646058

[B123] Schwanhausser B. Busse D. Li N. Dittmar G. Schuchhardt J. Wolf J. (2011). Global quantification of mammalian gene expression control. *Nature* 473 337–342. 10.1038/nature10098 21593866

[B124] Shi Y. Di Giammartino D. C. Taylor D. Sarkeshik A. Rice W. J. Yates J. R. III (2009). Molecular architecture of the human pre-mRNA 3′ processing complex. *Mol. Cell* 33 365–376. 10.1016/j.molcel.2008.12.028 19217410 PMC2946185

[B125] Smith C. W. Valcarcel J. (2000). Alternative pre-mRNA splicing: the logic of combinatorial control. *Trends Biochem. Sci.* 25 381–388. 10916158 10.1016/s0968-0004(00)01604-2

[B126] Sperling R. (2017). The nuts and bolts of the endogenous spliceosome. *Wiley Interdiscip. Rev. RNA* 8 e1377. 10.1002/wrna.1377 27465259

[B127] Squires J. E. Patel H. R. Nousch M. Sibbritt T. Humphreys D. T. Parker B. J. (2012). Widespread occurrence of 5-methylcytosine in human coding and non-coding RNA. *Nucleic Acids Res.* 40 5023–5033. 10.1093/nar/gks144 22344696 PMC3367185

[B128] Suter B. (2018). RNA localization and transport. *Biochim. Biophys. Acta Gene Regul. Mech.* 1861 938–951. 30496039 10.1016/j.bbagrm.2018.08.004

[B129] Taibi F. Metzinger-Le Meuth V. Massy Z. A. Metzinger L. (2014). miR-223: an inflammatory oncomiR enters the cardiovascular field. *Biochim. Biophys. Acta* 1842 1001–1009. 10.1016/j.bbadis.2014.03.005 24657505

[B130] Talati C. Sweet K. L. (2018). Nuclear transport inhibition in acute myeloid leukemia: recent advances and future perspectives. *Int. J. Hematol. Oncol.* 7:IJH04. 10.2217/ijh-2018-0001 30405902 PMC6219429

[B131] Tang C. Klukovich R. Peng H. Wang Z. Yu T. Zhang Y. (2018). ALKBH5-dependent m6A demethylation controls splicing and stability of long 3’-UTR mRNAs in male germ cells. *Proc. Natl. Acad. Sci. U.S.A.* 115 E325–E333. 10.1073/pnas.1717794115 29279410 PMC5777073

[B132] Tian B. Graber J. H. (2012). Signals for pre-mRNA cleavage and polyadenylation. *Wiley Interdiscip. Rev. RNA* 3 385–396. 10.1002/wrna.116 22012871 PMC4451228

[B133] Tollervey J. R. Wang Z. Hortobagyi T. Witten J. T. Zarnack K. Kayikci M. (2011). Analysis of alternative splicing associated with aging and neurodegeneration in the human brain. *Genome Res.* 21 1572–1582. 10.1101/gr.122226.111 21846794 PMC3202275

[B134] Tong J. Flavell R. A. Li H. B. (2018). RNA m(6)A modification and its function in diseases. *Front. Med.* 12:481–489. 10.1007/s11684-018-0654-8 30097961

[B135] Uil T. G. Haisma H. J. Rots M. G. (2003). Therapeutic modulation of endogenous gene function by agents with designed DNA-sequence specificities. *Nucleic Acids Res.* 31 6064–6078. 14576293 10.1093/nar/gkg815 PMC275457

[B136] Van Roosbroeck K. Calin G. A. (2016). MicroRNAs in chronic lymphocytic leukemia: miRacle or miRage for prognosis and targeted therapies? *Semin. Oncol.* 43 209–214. 10.1053/j.seminoncol.2016.02.015 27040698 PMC5533104

[B137] Viphakone N. Hautbergue G. M. Walsh M. Chang C. T. Holland A. Folco E. G. (2012). TREX exposes the RNA-binding domain of Nxf1 to enable mRNA export. *Nat. Commun.* 3:1006. 10.1038/ncomms2005 22893130 PMC3654228

[B138] Vu L. P. Pickering B. F. Cheng Y. Zaccara S. Nguyen D. Minuesa G. (2017). The N(6)-methyladenosine (m(6)A)-forming enzyme METTL3 controls myeloid differentiation of normal hematopoietic and leukemia cells. *Nat. Med.* 23 1369–1376. 10.1038/nm.4416 28920958 PMC5677536

[B139] Wahle E. Winkler G. S. (2013). RNA decay machines: deadenylation by the Ccr4-not and Pan2-Pan3 complexes. *Biochim. Biophys. Acta* 1829 561–570. 10.1016/j.bbagrm.2013.01.003 23337855

[B140] Wang K. Long B. Liu F. Wang J. X. Liu C. Y. Zhao B. (2016). A circular RNA protects the heart from pathological hypertrophy and heart failure by targeting miR-223. *Eur. Heart J.* 37 2602–2611. 10.1093/eurheartj/ehv713 26802132

[B141] Wein N. Vulin A. Findlay A. R. Gumienny F. Huang N. Wilton S. D. (2017). Efficient skipping of single exon duplications in DMD Patient-derived cell lines using an antisense oligonucleotide approach. *J. Neuromuscul. Dis.* 4 199–207. 10.3233/JND-170233 28869484

[B142] Weng H. Huang H. Wu H. Qin X. Zhao B. S. Dong L. (2018). METTL14 inhibits hematopoietic stem/progenitor differentiation and promotes leukemogenesis via mRNA m(6)A modification. *Cell Stem Cell* 22 191–205.e9. 10.1016/j.stem.2017.11.016 29290617 PMC5860916

[B143] Will C. L. Luhrmann R. (2011). Spliceosome structure and function. *Cold Spring Harb. Perspect. Biol.* 3:a003707. 10.1101/cshperspect.a003707 21441581 PMC3119917

[B144] Williams T. Ngo L. H. Wickramasinghe V. O. (2018). Nuclear export of RNA: different sizes, shapes and functions. *Semin. Cell Dev. Biol.* 75 70–77. 10.1016/j.semcdb.2017.08.054 28866329

[B145] Xia S. Feng J. Lei L. Hu J. Xia L. Wang J. (2016). Comprehensive characterization of tissue-specific circular RNAs in the human and mouse genomes. *Brief. Bioinform.* 18 984–992. 10.1093/bib/bbw081 27543790

[B146] Xing D. Liang J. Q. Li Y. Lu J. Jia H. B. Xu L. Y. (2014). Identification of long noncoding RNA associated with osteoarthritis in humans. *Orthop. Surg.* 6 288–293. 10.1111/os.12147 25430712 PMC6583210

[B147] Xu H. Guo S. Li W. Yu P. (2015). The circular RNA Cdr1as, via miR-7 and its targets, regulates insulin transcription and secretion in islet cells. *Sci Rep.* 5:12453. 10.1038/srep12453 26211738 PMC4515639

[B148] Yablonovitch A. L. Deng P. Jacobson D. Li J. B. (2017). The evolution and adaptation of A-to-I RNA editing. *PLoS Genet.* 13:e1007064. 10.1371/journal.pgen.1007064 29182635 PMC5705066

[B149] Yang H. Shang D. Xu Y. Zhang C. Feng L. Sun Z. (2017). The LncRNA connectivity map: using LncRNA signatures to connect small molecules, LncRNAs, and diseases. *Sci Rep.* 7:6655. 10.1038/s41598-017-06897-3 28751672 PMC5532316

[B150] Yang L. Huang P. Li F. Zhao L. Zhang Y. Li S. (2012). c-Jun amino-terminal kinase-1 mediates glucose-responsive upregulation of the RNA editing enzyme ADAR2 in pancreatic beta-cells. *PLoS One* 7:e48611. 10.1371/journal.pone.0048611 23139803 PMC3490865

[B151] Yang Y. Muzny D. M. Reid J. G. Bainbridge M. N. Willis A. Ward P. A. (2013). Clinical whole-exome sequencing for the diagnosis of mendelian disorders. *N. Engl. J. Med.* 369 1502–1511. 10.1056/NEJMoa1306555 24088041 PMC4211433

[B152] Yao Z. Luo J. Hu K. Lin J. Huang H. Wang Q. (2017). ZKSCAN1 gene and its related circular RNA (circZKSCAN1) both inhibit hepatocellular carcinoma cell growth, migration, and invasion but through different signaling pathways. *Mol. Oncol.* 11 422–437. 10.1002/1878-0261.12045 28211215 PMC5527481

[B153] Zhang Y. He Q. Hu Z. Feng Y. Fan L. Tang Z. (2016). Long noncoding RNA LINP1 regulates repair of DNA double-strand breaks in triple-negative breast cancer. *Nat. Struct. Mol. Biol.* 23 522–530. 10.1038/nsmb.3211 27111890 PMC4927085

[B154] Zhang Y. G. Yang H. L. Long Y. Li W. L. (2016). Circular RNA in blood corpuscles combined with plasma protein factor for early prediction of pre-eclampsia. *BJOG* 123 2113–2118. 10.1111/1471-0528.13897 26846540

[B155] Zhang Z. Tjian R. (2018). Measuring dynamics of eukaryotic transcription initiation: challenges, insights and opportunities. *Transcription* 9 159–165. 10.1080/21541264.2017.1363017 28920762 PMC5927711

[B156] Zheng D. Tian B. (2014). RNA-binding proteins in regulation of alternative cleavage and polyadenylation. *Adv. Exp. Med. Biol.* 825 97–127. 10.1007/978-1-4939-1221-6_3 25201104

[B157] Zheng G. Cox T. Tribbey L. Wang G. Z. Iacoban P. Booher M. E. (2014). Synthesis of a FTO inhibitor with anticonvulsant activity. *ACS Chem. Neurosci.* 5 658–665. 10.1021/cn500042t 24834807 PMC4140589

[B158] Zheng G. Dahl J. A. Niu Y. Fedorcsak P. Huang C. M. Li C. J. (2013). ALKBH5 is a mammalian RNA demethylase that impacts RNA metabolism and mouse fertility. *Mol. Cell* 49 18–29. 10.1016/j.molcel.2012.10.015 23177736 PMC3646334

[B159] Zheng Q. Bao C. Guo W. Li S. Chen J. Chen B. (2016). Circular RNA profiling reveals an abundant circHIPK3 that regulates cell growth by sponging multiple miRNAs. *Nat. Commun.* 7:11215. 10.1038/ncomms11215 27050392 PMC4823868

[B160] Zhou F. Qiu W. Yao R. Xiang J. Sun X. Liu S. (2013). CRM1 is a novel independent prognostic factor for the poor prognosis of gastric carcinomas. *Med. Oncol.* 30:726. 10.1007/s12032-013-0726-1 24026662

[B161] Zipeto M. A. Court A. C. Sadarangani A. Delos Santos N. P. Balaian L. Chun H. J. (2016). ADAR1 activation drives leukemia stem cell self-renewal by impairing Let-7 biogenesis. *Cell Stem Cell* 19 177–191. 10.1016/j.stem.2016.05.004 27292188 PMC4975616

